# Mental Health Help-Seeking Among Migrant and Domestic Workers in Singapore

**DOI:** 10.1192/j.eurpsy.2026.10983

**Published:** 2026-07-31

**Authors:** M. Subramaniam, Y. Zhang, P. Satghare, Y. S. Koh, A. Jeyagurunatha, S. Archana, C. Y. Lim, L. Azuree, H. Talib, S. A. Chong

**Affiliations:** 1 Research; 2 Institute of Mental Health; 3 Ministry of Manpower, Singapore, Singapore

## Abstract

**Introduction:**

Southeast Asia is home to intra- and inter-regional migrant workers (MWs), with the Asia–Pacific region hosting approximately 14.2% of them—around 24 million individuals, with many engaging in low-wage, labour-intensive, and often precarious employment. Migrant workers (MWs) and migrant domestic workers (MDWs) in Asia are at heightened risk of mental health problems due to long working hours, abuse, and family separation. In Singapore, despite existing support systems, formal help-seeking for mental health conditions remains limited.

**Objectives:**

The aims of the current study were to understand the mental health help-seeking behaviors among MWs and MDWs in Singapore using a mixed methods approach. The study quantitatively explored the workers’ awareness of organisations or agencies that they could seek help from; the people, agencies or organisations that they had sought help from; and the ways in which they would like information on mental health to be conveyed to them. A deeper understanding of their perceived barriers and facilitators to accessing care and the ways in which they had sought help for their emotional problems were qualitatively examined through focus group discussions (FGDs).

**Methods:**

A mixed-methods design was employed. Phase 1 was a quantitative survey involving 1,465 MWs and 1,462 MDWs from the six largest nationality groups in Singapore. Phase 2 comprised 14 focus group discussions with 48 MWs and 52 MDWs to explore their experiences in depth.

**Results:**

Most MWs (79.2%) and MDWs (91.4%) reported seeking help for emotional difficulties, mainly from informal sources such as family and friends. Awareness of formal services, including the Ministry of Manpower (MOM), was moderate (58.4% of MWs; 66.8% of MDWs). Both groups preferred face-to-face workshops for receiving mental health information. Help-seeking preferences diverged: 48.1% of MWs favored trained professionals, while 51.5% of MDWs preferred trained peers. Qualitative findings highlighted key barriers to be fear of job loss, financial constraints, limited awareness, and stigma, while facilitators included supportive family and friends, and positive, culturally sensitive healthcare encounters.

**Conclusions:**

Although many migrant workers seek support for emotional distress, reliance on informal networks persists due to structural and cultural barriers to formal care. Integrated support models incorporating peer-led approaches, culturally and linguistically competent services, and clearer communication about accessible resources are needed to strengthen mental health support for this population.

**Disclosure of Interest:**

None Declared

